# Dynamic Prediction of Maize Tasseling Stage Based on UAV LiDAR Time-Series Plant Height Growth Curves: A Framework Coupling UAV-CHM-POI

**DOI:** 10.3390/plants15152382

**Published:** 2026-08-03

**Authors:** Jixuan Yan, Kejing Cheng, Wenning Wang, Zichen Guo, Qiang Li, Jiaqin Yuan, Guang Li, Weiwei Ma, Yinshan Ma

**Affiliations:** 1 College of Water Conservancy and Hydropower Engineering, Gansu Agricultural University, No. 1 Yingmen Village, Anning District, Lanzhou 730070, China; chengkj@st.gsau.edu.cn (K.C.); wangwn@gsau.edu.cn (W.W.); guozichen1993@lzb.ac.cn (Z.G.); liqiang@lzb.ac.cn (Q.L.); 1073324020381@st.gsau.edu.cn (J.Y.); 2 Gansu Provincial Agricultural Smart Water-Saving Technology Innovation Center, Lanzhou 730070, China; 3 College of Agriculture and Ecological Engineering, Hexi University, Zhangye 734000, China; liguangyanjx@sina.com (G.L.); mays@hxu.edu.cn (Y.M.); 4 College of Forestry, Gansu Agricultural University, Lanzhou 730070, China; maww@gsau.edu.cn

**Keywords:** UAV LiDAR, maize plant height growth curve, tasseling stage identification, tasseling stage prediction

## Abstract

Accurate identification and effective prediction of the maize tasseling stage are of great significance for guiding precision field management and ensuring stable crop yields. Conventional manual observation methods suffer from high labor intensity, poor timeliness, and strong subjectivity. In this study, based on an unmanned aerial vehicle (UAV) remote sensing platform, LiDAR point cloud data and RGB imagery were simultaneously acquired to construct digital surface models (DSMs) and digital terrain models (DTMs). Multi-dimensional statistical features were extracted to establish a high-precision plant height estimation method applicable to the entire growth cycle of maize. On this basis, the Logistic growth curve function was introduced to fit the dynamic changes in plant height, enabling the identification and early prediction of the maize tasseling stage based on the plant height growth curve. The research results indicate the following: (1) For maize plant height estimation, the LiDAR sensor outperforms RGB. The optimal accuracy is achieved by combining the 99th percentile of DSM with the minimum DTM, yielding a root mean square error (RMSE) of 0.17 m. (2) Based on the high-accuracy plant height time series, the point of inflection (POI) achieves the highest accuracy in tasseling stage identification, with an RMSE of 2.586 d under the reconstructed time series. (3) Prediction accuracy of the tasseling stage improves with increasing plant height threshold, and optimal performance is observed when the threshold is ≥1.6 m with a growth rate between 0.11 and 0.13. This study establishes a technical framework of “time-series perception–dynamic simulation–feature identification–early prediction”, providing a scientific basis for automated monitoring and precision management of the maize tasseling stage. It holds significant theoretical and practical value for the advancement of smart agriculture and crop phenotyping research.

## 1. Introduction

Maize is the most widely planted and highest-yielding food crop in the world, serving as a crucial guarantee for national food security, a fundamental pillar of agricultural economic development, and a core raw material for feed and deep-processing industries [1]. With the upgrading of dietary structures, the growing demand for meat, eggs, and milk has driven sustained consumption of maize. China’s annual maize production is nearly 300 million tons, accounting for approximately 40% of the country’s total grain output [2]. However, increasing scarcity of arable land resources and a pronounced human-land conflict severely limit the potential for expanding cultivation area, making the improvement of per-unit yield increasingly urgent. Traditional extensive management practices are no longer adequate to meet the demands of modern precision agriculture [3]. Therefore, exploiting the potential for yield increase through refined field management and accurate monitoring of critical growth stages has become a pressing issue that urgently needs to be addressed.

Plant height is a core phenotypic parameter characterizing maize growth and development, with its dynamic changes spanning the complete life cycle from vegetative to reproductive stages [4]. This metric not only directly reflects the plant’s nutrient accumulation level and growth rate, but is also closely associated with lodging resistance, canopy structure, and light use efficiency [5]. Traditional plant height measurement relies on manual point-by-point sampling using rulers or leveling rods. Constrained by labor and time costs, only a few discrete time points can be obtained within a single growing season, making continuous monitoring of plant height dynamics difficult [6]. Furthermore, manual measurements are prone to subjective biases caused by differences in observer experience and sampling location, which compromise data consistency and reproducibility [7]. In recent years, the rapid development of unmanned aerial vehicle (UAV) remote sensing technology has provided significant technical support for high-throughput acquisition of crop phenotypic information. UAV platforms equipped with LiDAR or RGB cameras can rapidly acquire high-spatial-resolution canopy imagery and generate digital surface model (DSM) through 3D reconstruction algorithms, thereby enabling efficient and non-destructive estimation of plant height [8]. However, accurately extracting feature variables that reflect true plant height from massive remote sensing data remains a central challenge in current research. The traditional DSM-DTM differencing method is susceptible to interference from canopy internal gaps, point cloud noise, and ground vegetation cover, which limits the accuracy of plant height estimation. Therefore, optimizing plant height estimation methods to improve the accuracy and stability of effective information extraction in complex canopy scenes has become a key issue in applying UAV remote sensing technology to crop phenotyping [9]. Developing more refined data processing strategies and feature extraction algorithms is of great practical significance for fully leveraging the high-throughput observation advantages of UAVs and achieving precise monitoring of crop growth dynamics [9].

Accurate identification and prediction of critical crop phenophases are essential for agricultural production management and scientific research. As a vital indicator of crop life cycles, phenology reflects phased growth patterns and provides a scientific basis for agronomic practices such as sowing, fertilization, and irrigation. The tasseling stage in maize marks a pivotal transition from vegetative to reproductive growth, and its timing directly governs pollination efficiency and grain yield formation [10]. Tasseling time is a key component of maize flowering phenology and strongly influences local adaptation, reproductive synchrony, and yield formation. Its variation is therefore important for cultivar selection, germplasm evaluation, and breeding. Accurate, scalable monitoring of tasseling phenology can support the assessment of genotype-by-environment responses and the selection of hybrids with stable reproductive timing under variable climatic conditions. Traditional phenological monitoring relies heavily on manual field observations, typically defined as the point when over 50% of plants enter a specific growth stage. However, this method is labor-intensive, inefficient, and susceptible to subjective bias, making it inadequate for modern agriculture’s demand for large-scale, standardized phenological data [11]. While recent advancements in remote sensing offer efficient alternatives, current methods based on vegetation indices and spectral features-which infer phenological events from changes in canopy color or structure-face significant limitations [12]. These approaches often depend on high-quality optical imagery and are vulnerable to interference from atmospheric conditions, such as cloud cover and illumination variability. Furthermore, spectral information tends to saturate after canopy closure, reducing sensitivity in phenophase detection [13]. Plant height serves as a critical indicator of vegetative status and shares an intrinsic physiological link with the tasseling stage. Research indicates that tasseling typically coincides with the inflection point where plant height shifts from rapid growth to a plateau [14]. Consequently, identifying the tasseling stage based on the temporal dynamics of plant height offers a more direct and stable technical pathway for phenological monitoring. By characterizing the three-dimensional structural dynamics of plants rather than relying on indirect spectral inferences, this approach provides an objective reflection of physical growth processes and demonstrates superior robustness in complex field environments [15].

Based on these considerations, this study utilized a UAV-based multi-source remote sensing platform to systematically evaluate the suitability of various sensors for estimating maize plant height throughout the entire growth cycle, establishing a high-precision estimation framework. Furthermore, a Logistic growth model was introduced to fit the temporal dynamics of plant height, from which several morphological feature points were extracted to analyze and compare their accuracy and stability in identifying the tasseling stage. Building on this, a prediction method for the tasseling stage based on early-season height time-series was explored, identifying the optimal prediction thresholds and growth rate parameters for effective forecasting. These findings provide a theoretical basis and technical support for the precise monitoring of key maize phenophases and offer a scalable approach with potential applications in cultivar evaluation, high-throughput breeding, climate-resilient adaptation, and precision crop management.

## 2. Results and Analysis

### 2.1. Estimation of Maize Plant Height Using LiDAR and RGB Data

**Figure 1:**
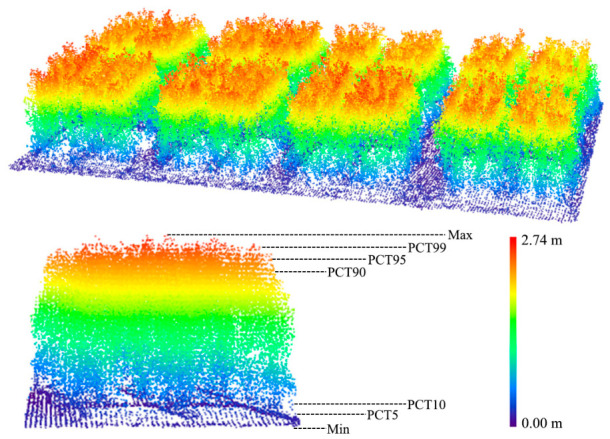
Side-view elevation distribution of maize point cloud.

To comprehensively characterize the distribution of canopy elevation, this study employed quantile-based metrics (Figure 1). Specifically, the Max represents the extreme uppermost point of the canopy, serving as an indicator of extreme plant height. The PCT99 and PCT95, as high-order quantiles, effectively reduce the influence of extreme outliers by truncating the tail of the distribution, thus providing a more robust estimation of the overall upper canopy level. The PCT90 and PCT10 capture the statistical distribution near the top and bottom of the canopy, respectively, thereby revealing the spatial differentiation of the canopy structure. Lastly, the Min denotes the lowest elevation value within the DSM or DTM, which generally corresponds to the ground surface or the base of the canopy.

**Figure 2:**
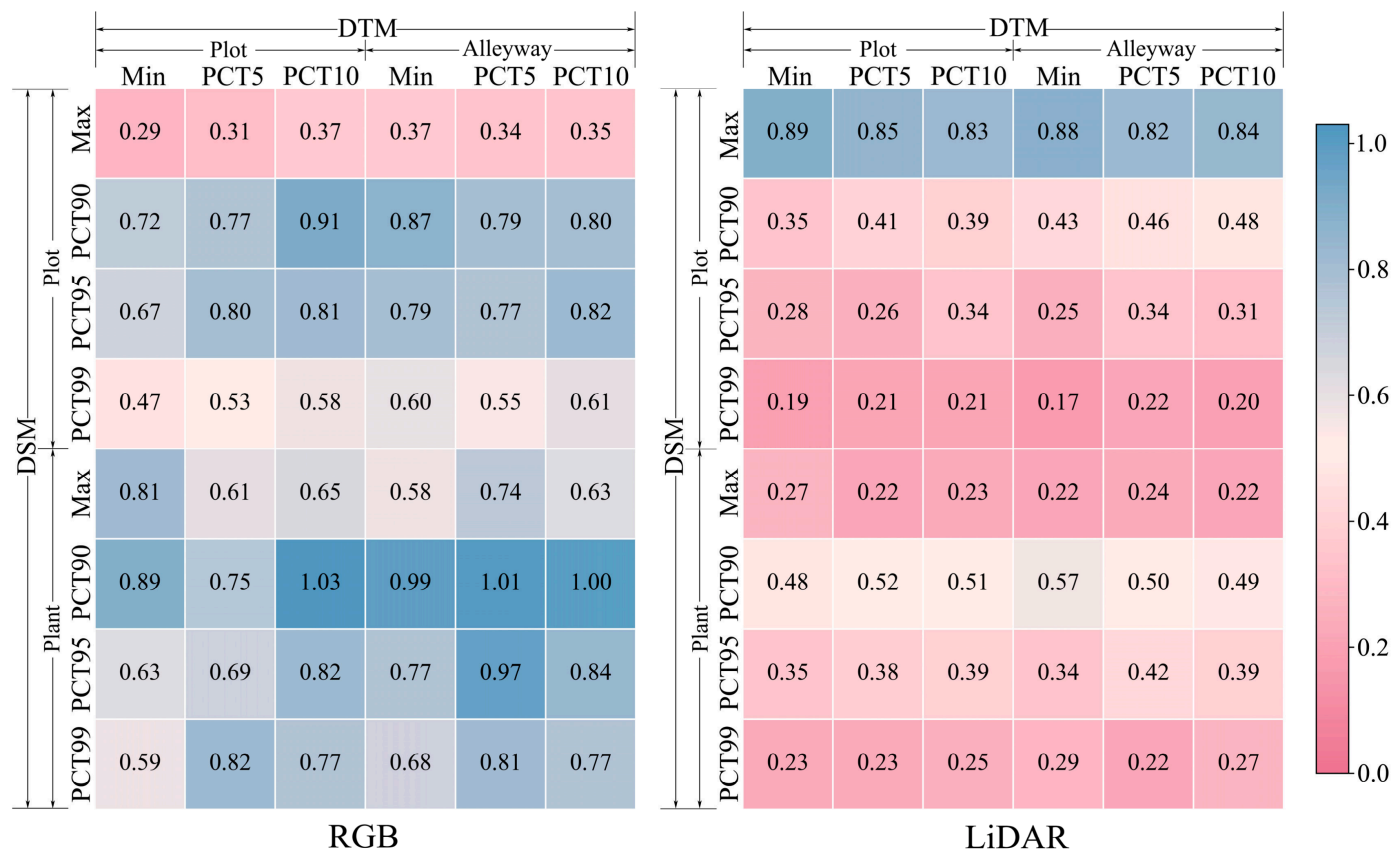
Screening for the optimal variable combination. Rows represent DSM-derived canopy-elevation variables, columns represent DTM-derived ground-elevation variables, and each cell shows the RMSE of the corresponding DSM-DTM combination for maize plant-height estimation. The left and right matrices represent the RGB- and LiDAR-derived results, respectively.

To systematically screen the optimal variable combinations for estimating maize plant height, this study conducted preliminary variable screening during the tasseling stage, when the canopy structure is most complex. As shown in Figure 2, the rows of each matrix represent candidate DSM canopy-top elevation metrics, whereas the columns represent candidate DTM ground-elevation metrics. The DSM metrics were extracted from either the plot-level ROI or the circular plant subregions, while the DTM metrics were extracted from either the plot-level ROI or the adjacent alleyway ground-reference region. Each cell reports the RMSE obtained by comparing field-measured plant height with the UAV-derived height calculated from the corresponding DSM–DTM combination. For the RGB sensor, using the DSM maximum value extracted from the plot-boundary ROI to represent the canopy top elevation yielded the best plant height estimation, with RMSE values ranging from 0.29 to 0.37 m across various ground variable combinations. Among these, the combination with the DTM minimum value extracted from the plot-boundary ROI achieved an RMSE of 0.29 m. In contrast, when high-percentile features derived from plot- or plant-subregion ROIs were used, the RMSE increased significantly to 0.47–1.03 m, with the mean statistical feature based on the plant-subregion ROI performing the worst. For the LiDAR sensor, the combination using the DSM maximum value extracted from the plot-boundary ROI resulted in a relatively higher RMSE, ranging from 0.82 to 0.89 m. Conversely, when high-percentile features derived from plot- or plant-subregion ROIs were applied, the RMSE decreased markedly to 0.17–0.57 m. Among these, the combination of the PCT99 from the plot-boundary ROI and the DTM minimum value performed best, achieving an RMSE as low as 0.17 m.

**Figure 3:**
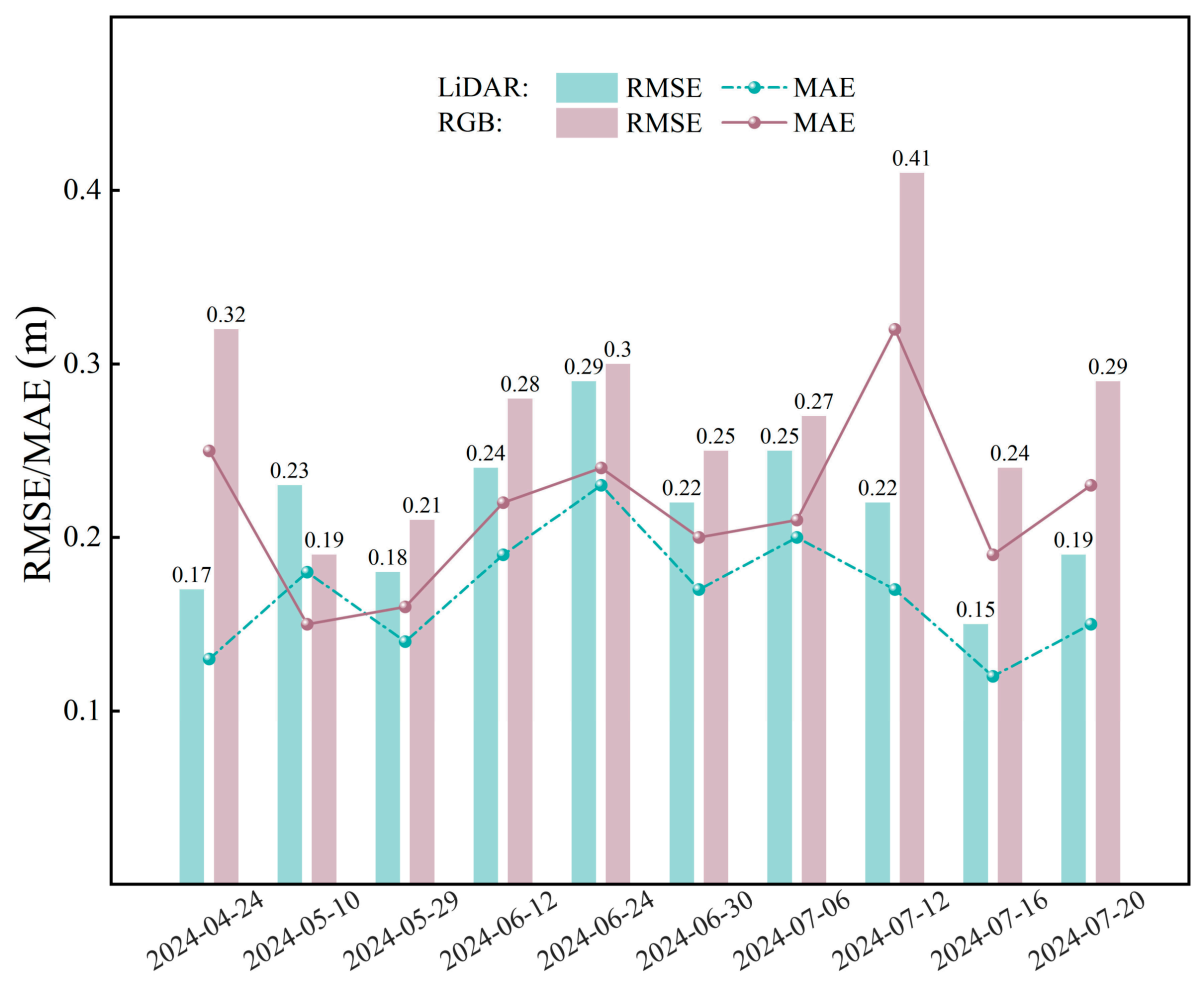
Estimation accuracy of plant height using different sensors.

As shown in Figure 3, the LiDAR sensor outperforms the RGB sensor in estimating plant height during most periods. The RMSE and MAE for LiDAR range from 0.15 to 0.29 m and from 0.12 to 0.23 m, respectively, while those for RGB range from 0.19 to 0.41 m and from 0.15 to 0.32 m, respectively. This indicates that LiDAR has better temporal stability, particularly during the middle and late growth stages of maize. From 12 July to 20 July, the RMSE of LiDAR remains below 0.22 m, whereas that of RGB fluctuates between 0.24 and 0.41 m.

### 2.2. Tasseling Stage Identification in Maize Based on Plant Height Time Series Across the Full Growth Period

**Figure 4:**
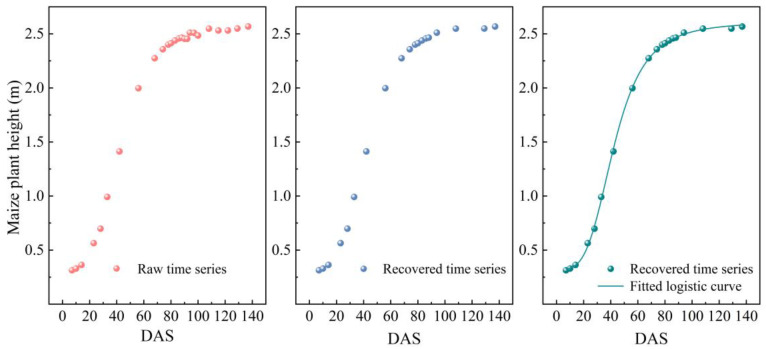
Recovered maize plant height time series over the full growth period.

Maize plant height estimated from UAV-based remote sensing is defined as the difference between the canopy top elevation and the ground elevation. This estimation is susceptible to external disturbances such as lodging and strong winds, which can produce anomalously low values that fail to reflect the actual growth dynamics. To mitigate the impact of such anomalous values on time-series analysis, this study proposes a plant height time-series reconstruction method based on the biological assumption that under normal conditions, maize plant height follows a monotonic increase. After excluding outliers, a growth model is fitted to the observed values to generate a continuous reconstructed series. Figure 4 illustrates the original observations, the reconstructed series, and the fitted curve for a representative plot. The results show that the reconstructed series and the fitted curve are in close agreement at each time point, fully preserving the original growth trend. This approach not only eliminates abnormal fluctuations caused by environmental factors but also transforms the plant height time-series data from discrete observations into a continuous curve.

**Figure 5:**
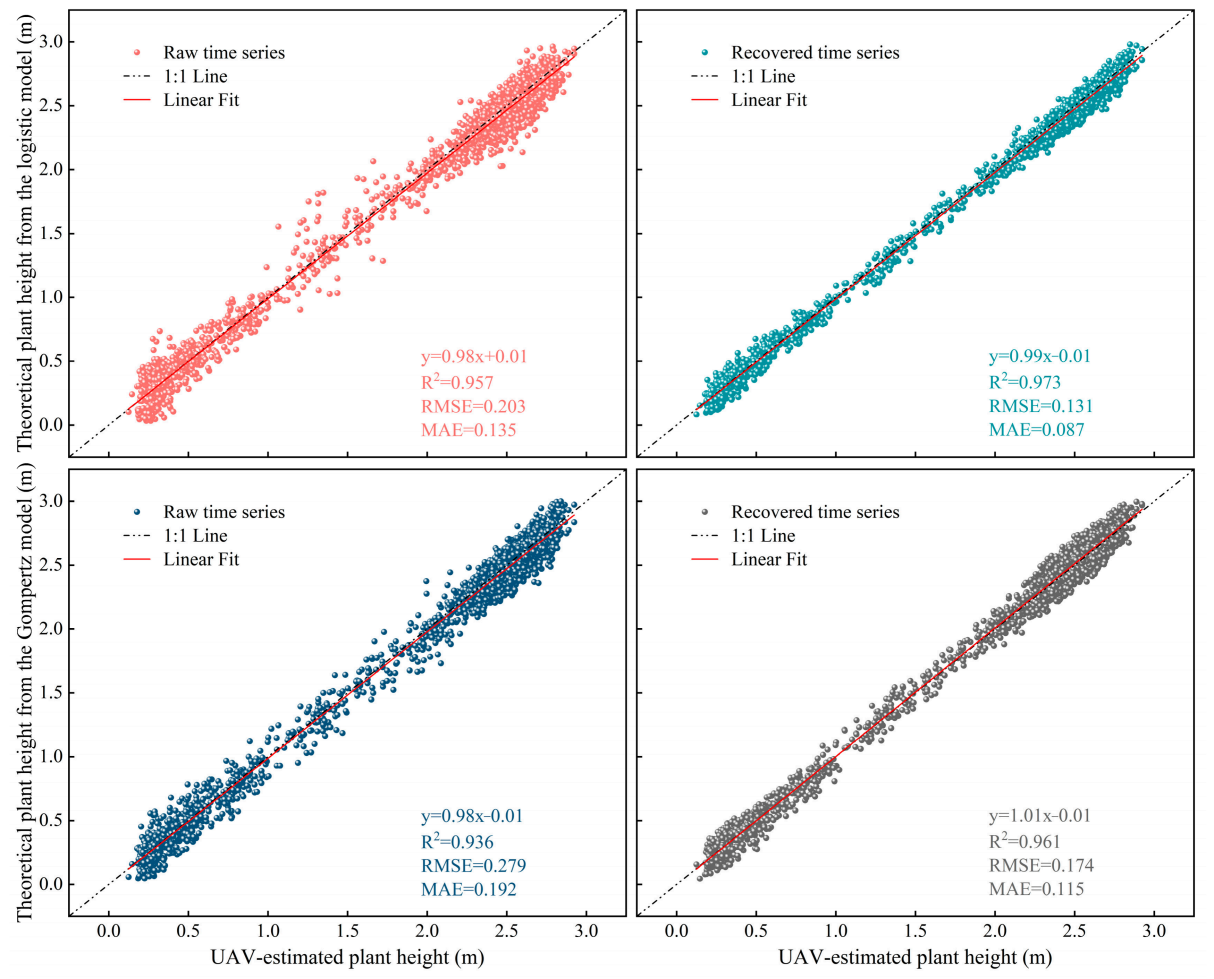
Comparison of consistency between UAV-estimated and theoretical plant height values before and after time-series recovery.

The consistency between the maize plant height time series estimated by UAV remote sensing and the growth functions serves as the theoretical premise and methodological basis for subsequent identification of the tasseling stage based on characteristic points of the growth curve. To quantitatively describe the dynamic pattern of plant height over time, this study fitted the plant height time series using the Logistic and Gompertz models (Figure 5). Unknown parameters in the models were solved by the least squares method, yielding a continuous theoretical curve that characterizes the growth process of maize plant height, thereby achieving a transformation from discrete observations to a continuous function.

Anomalous low values in the raw plant height sequence disrupted the fitting of the Logistic model, yielding an R^2^ of 0.957 and an RMSE of 0.203 m. Following the removal of outliers, the scatter points were tightly clustered around the 1:1 reference line, with R^2^ improving to 0.973 and RMSE decreasing to 0.131 m, a reduction of 35.4% compared to the raw sequence. For the Gompertz model, the raw sequence gave an R^2^ of 0.936, an RMSE of 0.279 m, and an MAE of 0.192 m, indicating slightly lower accuracy than the Logistic model, with an RMSE that was 37.4% higher. Post-reconstruction, all performance metrics improved substantially: R^2^ increased to 0.961, RMSE fell to 0.174 m, a 37.6% reduction, and MAE decreased to 0.115 m. However, compared to the reconstructed Logistic model, the Gompertz model still showed a lower R^2^ by 0.012 and a higher RMSE by 32.8%. Both models achieved pronounced and comparable improvements in accuracy after anomaly removal, demonstrating the broad applicability of the outlier removal method founded on the monotonic increase assumption across different growth models.

After comparison, the Logistic model outperformed the Gompertz model in describing maize plant height dynamics. Therefore, it was selected as the basis for tasseling stage identification. Analysis of the fitted parameters shows that the recovered time series had a mean plant height growth rate of 0.1180, slightly lower than the 0.1189 of the raw time series, but the standard deviation decreased from 0.0244 to 0.0184, indicating less variability (Table 1). The mean maximum plant height increased from 2.537 m to 2.670 m, with similar standard deviations.

**Figure 6:**
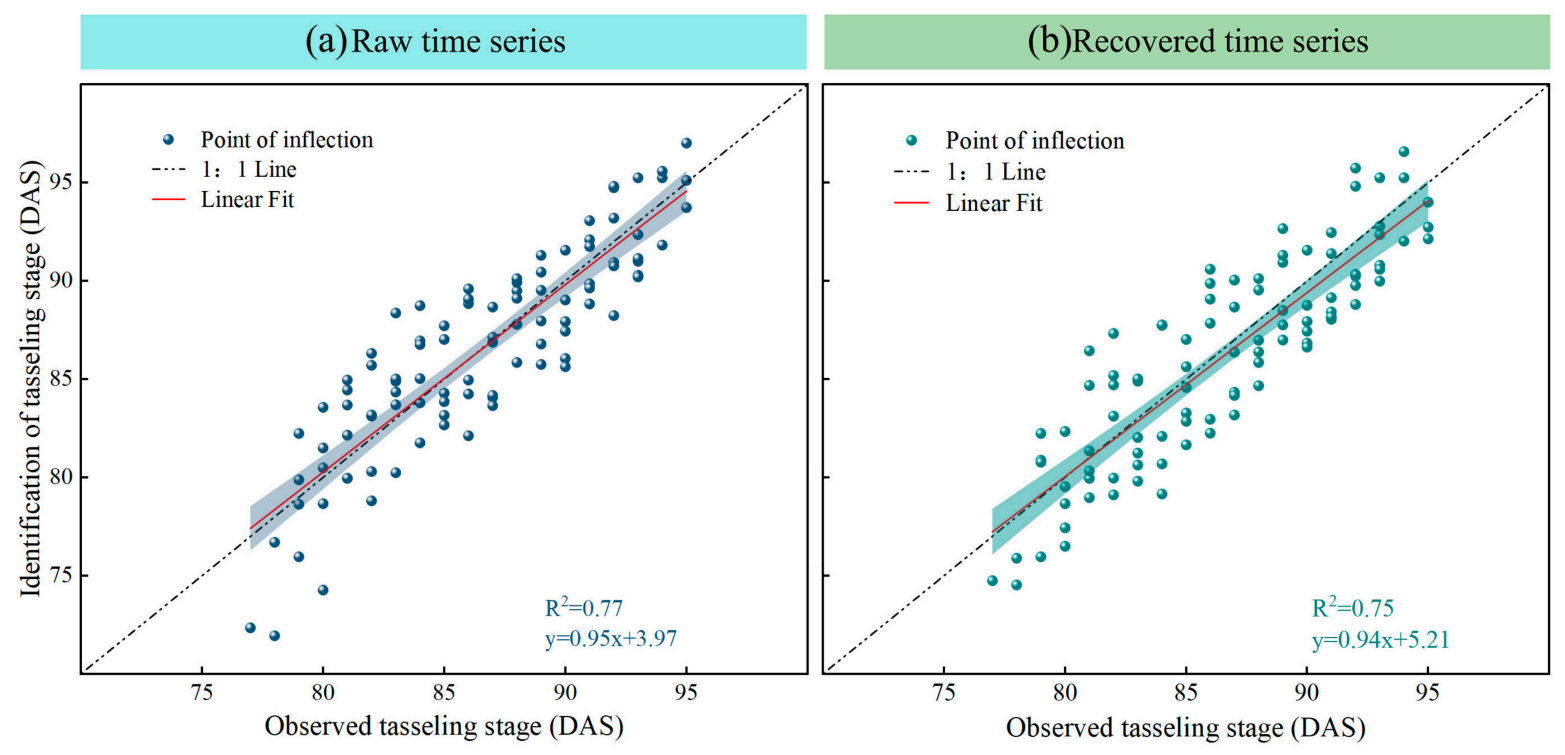
Tasseling stage identified by inflection point.

To achieve automatic identification of the maize tasseling stage based on the plant height growth curve, this study extracted five feature points closely related to changes in growth curve morphology as criteria for identifying the tasseling stage. The growth curves were fitted using both the raw plant height time series and the recovered plant height time series, and the aforementioned feature points were extracted to determine the tasseling date. The identification results were then compared with field-observed tasseling dates to evaluate the accuracy and reliability of each feature point. The results showed that the inflection point exhibited the best identification accuracy, as shown in Figure 6, with R^2^ values ranging from 0.75 to 0.77, and demonstrated strong adaptability across different experimental conditions, with small variation in R^2^.

Table 2 presents the error evaluation results for identifying maize tasseling stage based on different feature points. Overall, there are significant differences in identification accuracy among the feature points. In the raw time series, the inflection point achieved the highest accuracy with an RMSE of 2.478 days and an MAE of 2.124 days. After recovery, its error increased slightly but remained the lowest. The point of minimum curvature ranked second, with an RMSE of 3.909 days in the raw time series, which rose markedly to 5.356 days after recovery. The point of third derivative peak fell in the middle, with an RMSE of 4.385 days in the raw time series, decreasing to 3.542 days after recovery, indicating improved accuracy. The point of growth rate threshold and the point of normalized threshold showed relatively large errors, with raw RMSE values of 5.961 days and 5.897 days, respectively. After recovery, the former decreased while the latter increased, suggesting that different feature points respond differently to data smoothing.

**Figure 7:**
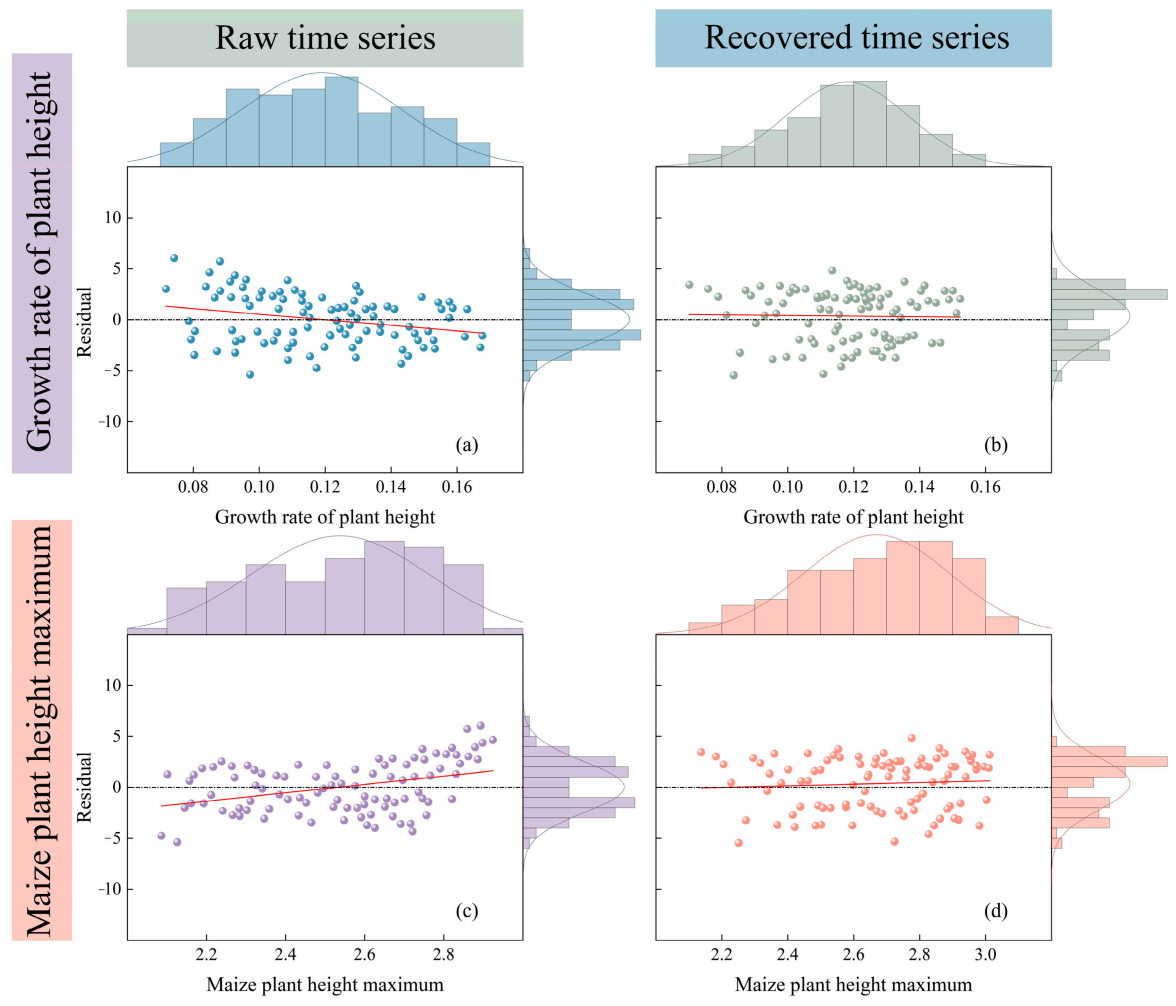
Residual distribution of tasseling stage identification: (**a**) original time-series plant height growth rate; (**b**) reconstructed time-series plant height growth rate; (**c**) original time-series maximum plant height; (**d**) reconstructed time-series maximum plant height.

Figure 7 shows the association distribution between residuals of tasseling stage identified by the inflection point method and parameters of the plant height growth curve across multiple experimental treatments in 2024. In the raw time series, the residuals of tasseling stage identification based on the inflection point were randomly distributed around zero, with balanced positive and negative deviations as plant height growth rate varied, showing no systematic bias. The residual distribution of the recovered time series was consistent with that of the raw time series, and the fitted line tended to be smoother. The proportions of positive and negative residuals were essentially balanced in both the raw and recovered time series, with no inherent tendency for systematic lead or lag. This indicates that the errors in inflection point identification were primarily caused by random factors rather than systematic biases induced by changes in growth curve parameters.

### 2.3. Prediction of the Maize Tasseling Stage Using Early-Stage Plant Height Time Series

To evaluate the cross-year transferability of the proposed prediction method, the 2024 growing season was treated as the source year for prior-parameter estimation, whereas the 2025 growing season was treated as the target year for prediction evaluation. Both growing seasons were conducted at the same experimental site using the same maize hybrids, planting-density treatments, and experimental design. The mean growth-rate parameter derived exclusively from the full-season plant-height time series collected in 2024 was transferred to the Logistic model and fixed at 0.119 for the prediction of tasseling dates in 2025.

Given the limited number of early-season observations, simultaneous estimation of both the growth-rate parameter and maximum plant height may increase parameter uncertainty and reduce extrapolation stability. Therefore, the growth-rate parameter was transferred from the source year and fixed during prediction. In contrast, maximum plant height was retained as a plot-specific parameter and estimated separately for each plot using the available 2025 observations. Maximum plant height may vary among hybrids, planting-density treatments, and growing seasons; fixing it at a common value derived from 2024 would impose the same asymptotic height on all plots and could introduce systematic prediction bias.

**Figure 8:**
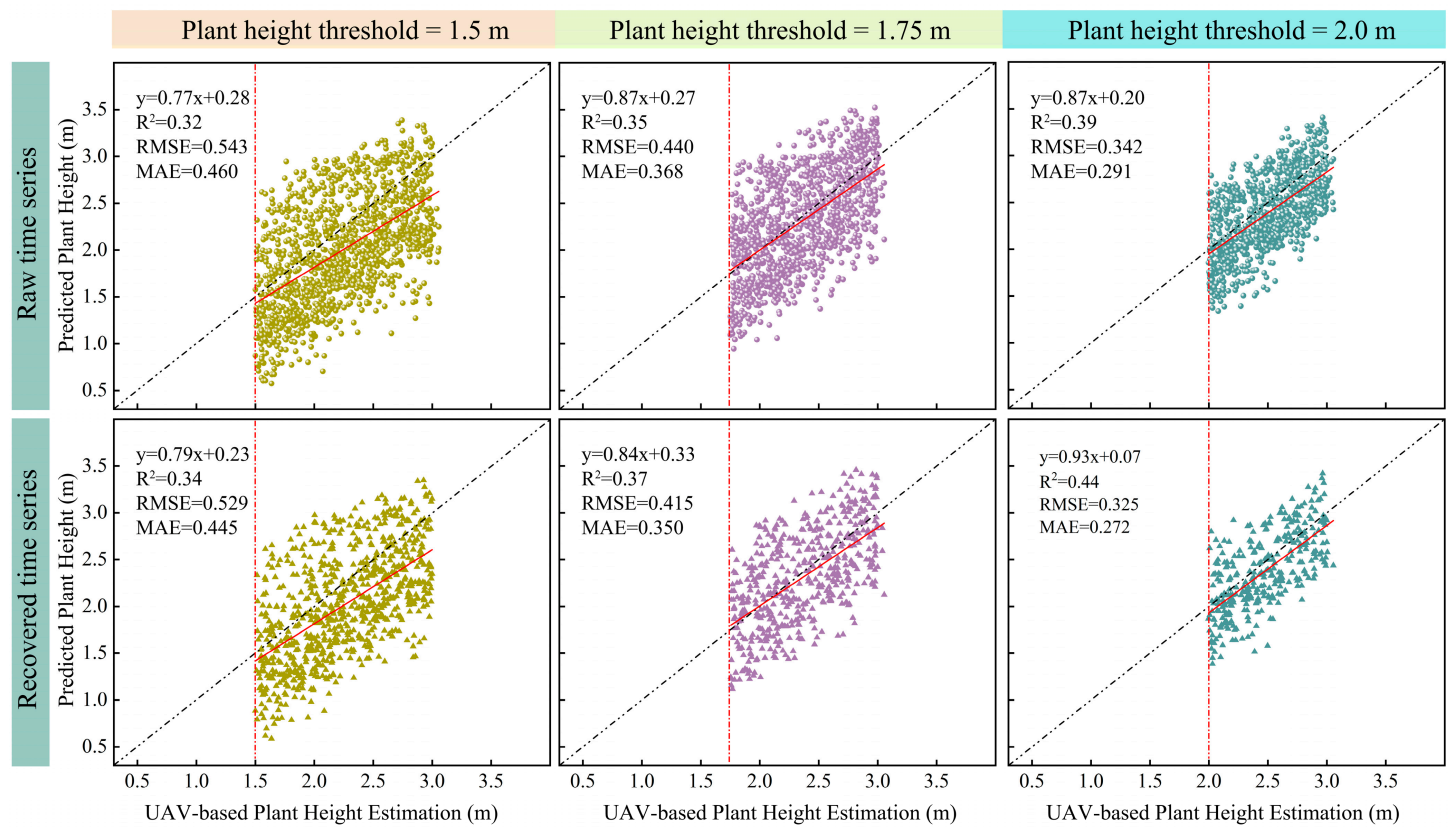
Prediction performance of raw and recovered plant height time series under different thresholds.

To investigate the effect of prediction initiation timing on the accuracy of maize tasseling stage prediction, three plant height thresholds of 1.5 m, 1.75 m, and 2.0 m were set, corresponding to stages of limited information, intermediate node, and sufficient information, respectively, to reveal the trade-off between prediction accuracy and initiation timeliness. As shown in Figure 8, as the threshold increased from 1.5 m to 2.0 m, the RMSE and MAE of plant height prediction showed a steady decreasing trend. Under the raw time series, the RMSE at the 1.5 m threshold was 0.543 m, where the data only covered the first half of the rapid growth period, leading to large extrapolation uncertainty. At the 1.75 m threshold, the RMSE decreased to 0.440 m. At the 2.0 m threshold, the RMSE further improved to 0.342 m, with the modeling data extending to near the inflection point, allowing the model to capture the growth trend more accurately. Across the three threshold levels, prediction accuracy based on the recovered time series was better than that based on the raw series. At the 1.5 m threshold, the RMSE decreased from 0.543 m to 0.529 m, an accuracy improvement of approximately 2.58%. As the threshold increased from 1.5 m to 2.0 m, the RMSE of the raw series decreased by 0.201 m, and that of the recovered series decreased by 0.204 m, indicating that increasing data volume is the primary factor for improving accuracy. Even after recovery, the prediction error at the 1.5 m threshold remained higher than the error of the raw series at the 2.0 m threshold, suggesting that data volume plays a more decisive role in accuracy than data quality.

**Figure 9:**
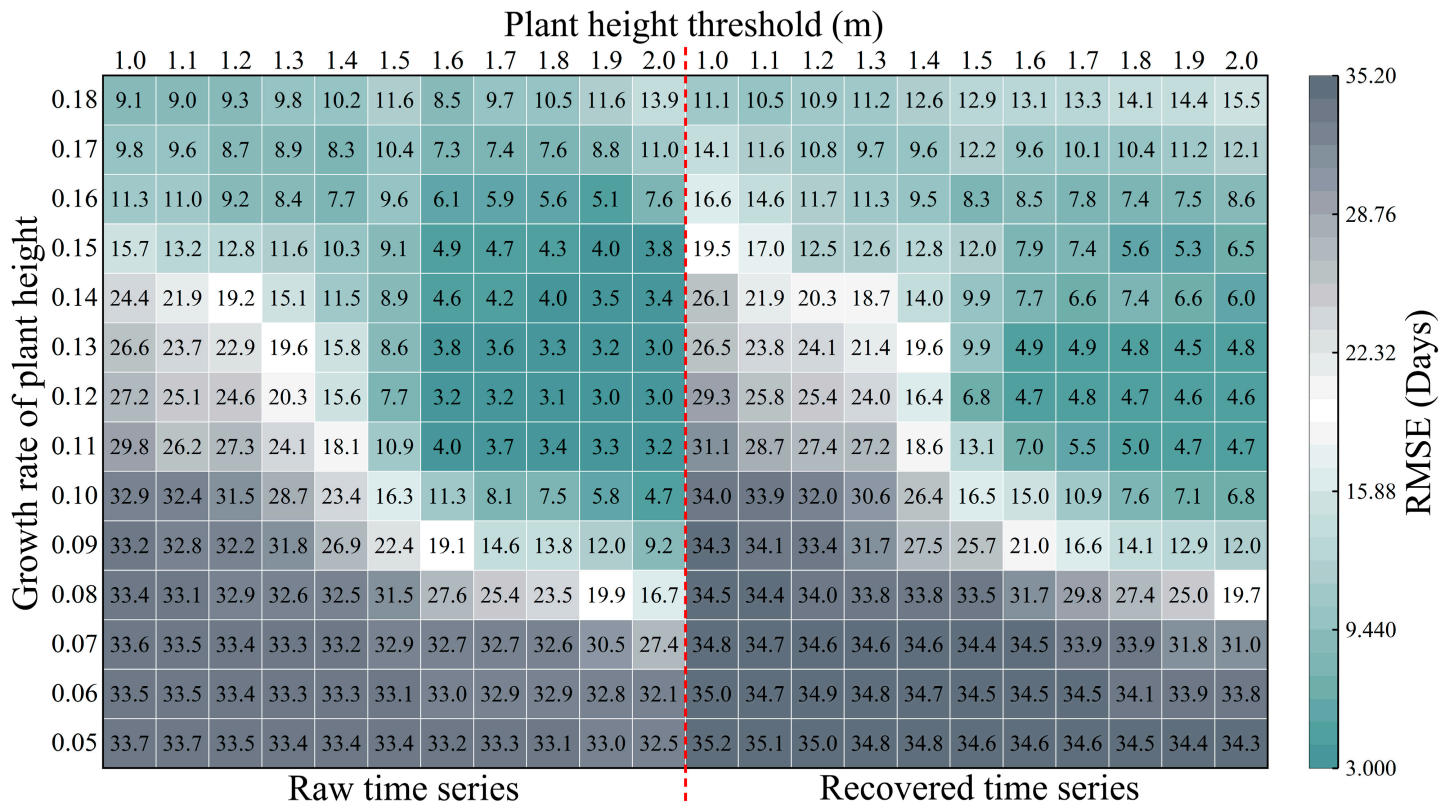
Prediction accuracy of maize tasseling stage under different combinations of plant height threshold and growth rate.

To systematically investigate the combined effects of plant height threshold and growth rate on tasseling stage prediction accuracy, a grid search approach was employed with plant height threshold ranging from 1.0 to 2.0 m at 0.1 m intervals and growth rate ranging from 0.05 to 0.18 at 0.01 intervals, resulting in 154 parameter combinations. RMSE was used as the evaluation metric. The results presented in Figure 9 indicate that under the raw time series, when the plant height threshold was at least 1.6 m and the growth rate fell within the 0.11–0.13 interval, the RMSE remained stable between 3.0 and 4.0 days. At a threshold of 1.6 m and a growth rate of 0.12, the RMSE was 3.2 days. When the growth rate deviated to 0.10, the RMSE increased to 11.3 days; when it increased to 0.14, the RMSE rose to 4.6 days. For the recovered time series, the optimal RMSE range was between 4.5 and 7.0 days. When the plant height threshold was below 1.3 m, the RMSE consistently exceeded 20 days even at the optimal growth rate, suggesting that the model struggled to capture growth characteristics due to insufficient early-stage data. When the threshold was raised to 2.0 m with a growth rate of 0.12, the RMSE dropped to 3.0 days for the raw time series, with a notably smaller error increase compared to the low-threshold scenarios.

**Figure 10:**
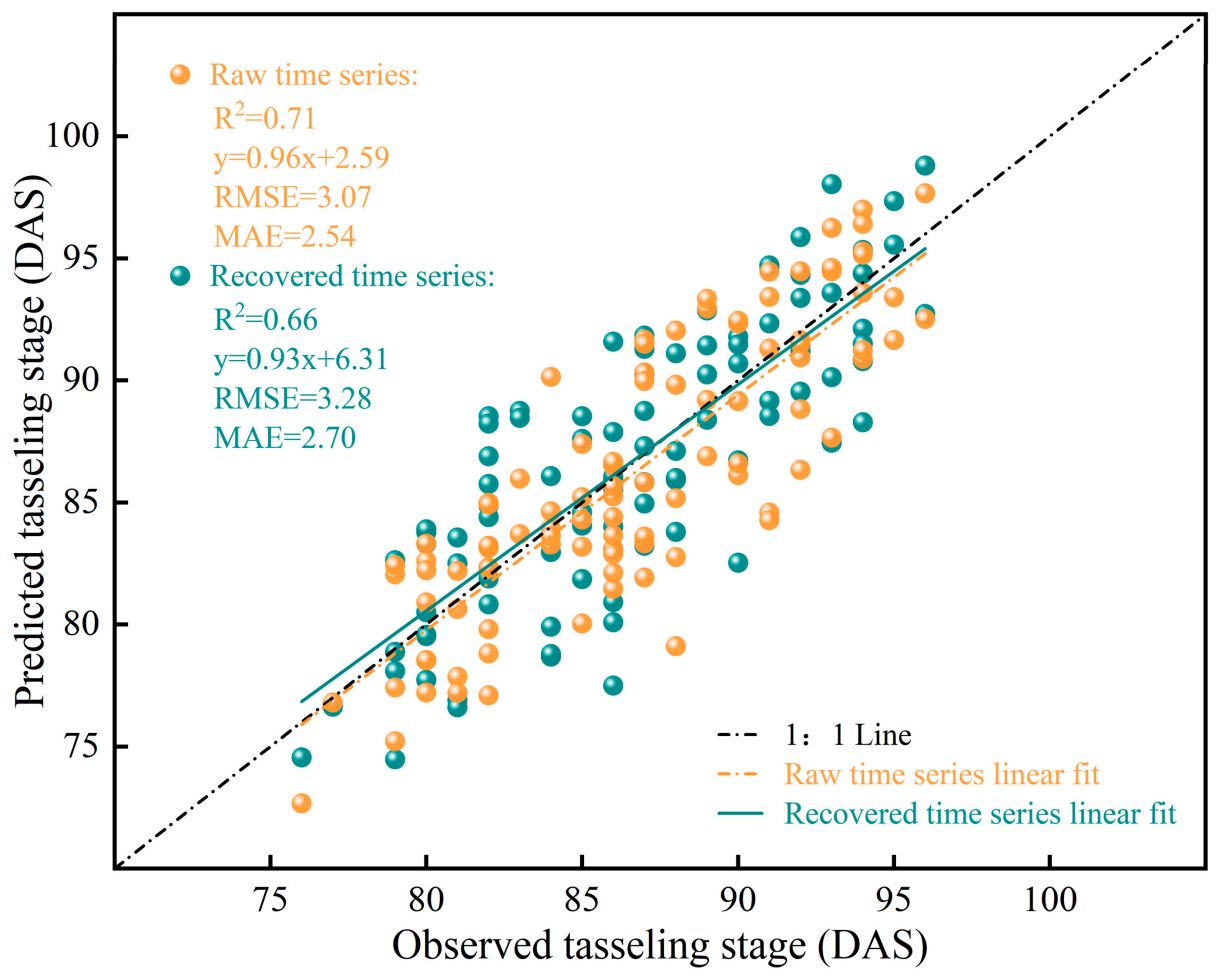
Comparison of tasseling stage prediction accuracy using original vs. recovered plant height time series.

In this study, the plant height growth rate was fixed at 0.119, the prediction initiation threshold was set at 1.6 m, and the tasseling stage of maize in 2025 was predicted based on the inflection point of the extrapolated growth curve of plant height. Overall, predicted values under both the raw and the recovered time series exhibited a good linear relationship with observed values, with data points closely scattered around the 1:1 line. According to the accuracy statistics presented in Figure 10, the MAE and RMSE of the prediction based on raw data were 2.54 days and 3.07 days, respectively. For the recovered data, the MAE and RMSE were 2.70 days and 3.28 days, respectively, indicating a slight decrease in accuracy after recovery, with increases of 0.16 days in MAE and 0.21 days in RMSE. These results suggest that early-season plant height time series recovery did not significantly improve the accuracy of tasseling stage prediction.

## 3. Discussion

### 3.1. Variations in the Stability and Accuracy of Different Sensors for Estimating Maize Plant Height Across the Full Growth Period

This study aimed to develop a method based on UAV remote sensing for accurately and reliably estimating maize plant height across multiple experimental conditions and growth stages. Using recovered plant height time series, the study further identified and predicted the date of maize tasseling. The results showed that the LiDAR sensor achieved higher estimation accuracy and greater temporal stability throughout the growth cycle. In contrast, the performance of the RGB sensor was more susceptible to growth stage and environmental conditions, exhibiting a wider range of estimation errors compared to LiDAR. These findings provide methodological support for identifying tasseling stages based on recovered plant height time series and offer a reference for evaluating the applicability of multi-sensor systems in crop phenotyping research. The field and UAV observations were spatially aligned by retaining the non-destructive plant-height sampling area within the UAV ROI and excluding destructive-sampling, edge, missing-plant, lodging, and weed-affected areas. Nevertheless, the field reference represented the mean height of three to six sampled plants, whereas the UAV-derived metric summarized upper-canopy structure over a larger plot-level area. The reported estimation errors should therefore be interpreted as the empirical correspondence between plant-level field sampling and plot-level canopy observations. The variable and limited field sample size may not fully characterize the complete plant-height distribution within each plot. Future studies should adopt a larger and fixed number of randomly or systematically selected plants and collect a more complete within-plot height distribution to support matched comparisons with UAV-derived maximum and percentile metrics and to quantify sampling uncertainty.

The primary reason for the high accuracy of LiDAR lies in its active imaging characteristics. By emitting pulsed signals and recording the return time, LiDAR directly acquires three-dimensional coordinate information, which is not affected by changes in illumination conditions and can function properly in low-light or even nighttime environments [16]. Moreover, LiDAR pulses have inherent penetrating capabilities, enabling them to pass through canopy gaps and capture return signals from within the canopy and even the ground. Combined with point cloud classification algorithms, ground points can be effectively separated from vegetation points in the mixed point cloud, thereby constructing a more accurate DTM [17]. Malambo et al. [18] demonstrated that quantile-based statistical features of point clouds can effectively characterize canopy height distribution and exhibit strong robustness to outlier noise. The findings of this study further validate the applicability of this conclusion throughout the entire maize growth cycle. Tunca et al. [19] used the SfM algorithm to estimate sorghum plant height by generating 3D models from high-resolution multispectral images. By comparing these models with field-measured data, they confirmed the high accuracy of the SfM algorithm in crop height estimation, reporting an R^2^ of 0.92 and demonstrating considerable application potential. Wang Huanchen et al. [20] adopted this same method in their study on plant height estimation of soybean and maize under an intercropping system. By comparing a DTM generated using mean interpolation with one generated using linear interpolation, combined with a DSM obtained from UAV imagery to construct a canopy height model, the results indicated that the DTM generated by linear interpolation yielded superior estimation performance, with an R^2^ of 0.85. This confirmed the feasibility of constructing a CHM via interpolation for plant height estimation in the absence of complete DTM data. Collectively, the studies reviewed above fully demonstrate that the CHM construction approach based on DSM and DTM statistical features has matured into a robust technical framework for crop height estimation, thereby providing a solid foundation for the methodology used in this research to estimate maize plant height. It should be noted that the UAV-derived canopy height represents the upper-canopy structure at the plot scale, whereas the field reference represents the mean height of 3–6 sampled plants. Therefore, the two measurements are not strictly equivalent, and their differences in spatial scale and statistical definition may contribute to the estimation error.

### 3.2. Effect of Maize Plant Height Growth Curve Fitting and Recovered Time Series on Tasseling Stage Identification

The consistency between the maize plant height time series estimated by UAV remote sensing and the growth function serves as the theoretical premise and methodological basis for identifying the tasseling stage based on characteristic points of growth curves. In this study, the Logistic and Gompertz models were respectively used to fit the plant height time series data. Research shows that the Logistic model can effectively characterize the cumulative growth process of maize plant height, with a goodness-of fit exceeding 97%. Wardhani et al. [21] compared the performance of the Logistic and Gompertz models in fitting maize plant height and evaluated their applicability in describing the maize growth process. Their results showed that the Logistic model achieves higher fitting accuracy in describing the cumulative growth process of maize plant height, particularly excelling during the early and middle growth stages, whereas the Gompertz model is more suitable for describing changes in growth rate. Similarly, Zhang Yuhong et al. [22] confirmed that the Logistic model is one of the optimal models for describing the dry matter accumulation process in maize, effectively capturing the cumulative dynamics of aboveground dry matter over days of growth.

The logistic model provided a more stable characterization of plant height dynamics in the recovered time series, with significantly smaller deviations between discrete observations and the fitted curve. This indicates that the recovery process effectively removed random noise from the original data, leading to more robust parameter estimation of the growth curve. Previous studies have also confirmed that smoothing recovery of crop time series data can significantly improve the goodness of fit of growth models. Zhang et al. [23] showed that reconstruction methods such as Savitzky–Golay filtering achieve high fitting accuracy for NDVI time series data, with strong performance in metrics such as RMSE and R^2^. Similarly, Zhao et al. [24] demonstrated that an adaptive denoising method could substantially reduce data noise while preserving vegetation change details, thereby enhancing the performance of growth models. As a result, the recovery process aligns the growth curve more closely with the inherent growth patterns of the crop. Local fluctuations in the original time series due to observation errors were effectively suppressed, making the S-shaped pattern of the curve more pronounced. Estimates of the plant height growth rate and maximum plant height consequently became more consistent with their true biological values.

### 3.3. Impact of Plant Height Threshold on the Accuracy of Predicting Maize Tasseling Stage

This study used a time series of maize plant height across the full growth period, acquired via UAV-based LiDAR, to systematically investigate the combined effects of prediction initiation timing and plant height growth rate on the accuracy of tasseling stage prediction under three different plant height thresholds. The results show that the determination of the plant height threshold is a key factor influencing early prediction accuracy. Prediction accuracy improved significantly with an increase in the threshold, and while the recovery process optimized prediction performance to a certain extent, it could not fully compensate for the information deficit caused by insufficient early data. In terms of the variation in prediction accuracy with increasing thresholds, when the plant height threshold was raised from 1.5 m to 2.0 m, the RMSE of the raw time series decreased from 0.543 m to 0.342 m, representing a reduction of 37.0%. This result highlights the important influence of the amount of early observational data on the extrapolation capability of the logistic model. When the threshold was set to 1.5 m, the early data used for modeling covered only the first half of the rapid plant height increase. At this stage, features near the curve inflection point were not yet fully apparent, making it difficult for the model to accurately estimate subsequent growth rates and maximum plant height. This led to greater uncertainty in extrapolation. As the threshold was raised to 2.0 m, the modeling data extended to the region approaching the inflection point of the growth curve. The model was then able to more accurately capture the trend of the growth rate, and consistency between the predicted and observed values improved significantly. Anderson et al. [25] used UAV-based phenotyping techniques to study maize growth trajectories and similarly found that fitting plant height data with sigmoidal growth functions yielded high accuracy, with the inflection point being highly correlated with flowering time, indicating that phenological prediction based on growth curves has a solid theoretical foundation. The present study further found that increasing the plant height threshold from 1.5 m to 1.75 m reduced the RMSE by 0.103 m, while raising it further from 1.75 m to 2.0 m reduced the RMSE by an additional 0.098 m. The magnitude of reduction was essentially consistent across both intervals. This trend indicates that before plant height reaches approximately 60–70% of its final value, each additional increment of early observational data contributes a relatively stable improvement in model accuracy. Liu et al. [15] used UAV-based LiDAR data to predict the maize tasseling stage and found that optimal prediction results were achieved when canopy height exceeded half of the maximum height. They also noted that tasseling stage prediction accuracy increases with a longer plant height time series, which aligns closely with the threshold effect observed in this study.

The prediction analysis in this study should be interpreted as a cross-year transfer experiment rather than a random validation based on pooled data from the two growing seasons. The 2024 dataset was used as the source year for prior-parameter estimation, whereas the 2025 dataset was used as the target year for prediction evaluation. Both experiments were conducted at the same site using the same maize hybrids, planting-density treatments, experimental design, and field-management practices. The use of DAS provided a consistent temporal scale for the same-site experiments but did not explicitly account for thermal accumulation. Therefore, the transferability of the fitted parameters across contrasting climatic environments or planting dates should be interpreted with caution. Future studies will compare DAS- and growing degree day (GDD)-based growth models and integrate temperature, solar radiation, precipitation, and soil variables to improve the biological relevance and environmental transferability of the framework. Compared with data-driven deep temporal models, the present framework emphasizes biological interpretability and applicability under limited sample conditions. However, its comparative performance against these models requires further evaluation using larger datasets. The proposed UAV-based framework may also support high-throughput evaluation of tasseling responses across maize hybrids and environments, providing useful phenotypic information for cultivar selection and climate-resilient breeding. Early tasseling prediction could further improve the timing of irrigation, nutrient management, and other field operations. However, these broader applications require validation across additional years, locations, and environmental conditions.

## 4. Data Sources

### 4.1. Extent of the Study Area

**Figure 11:**
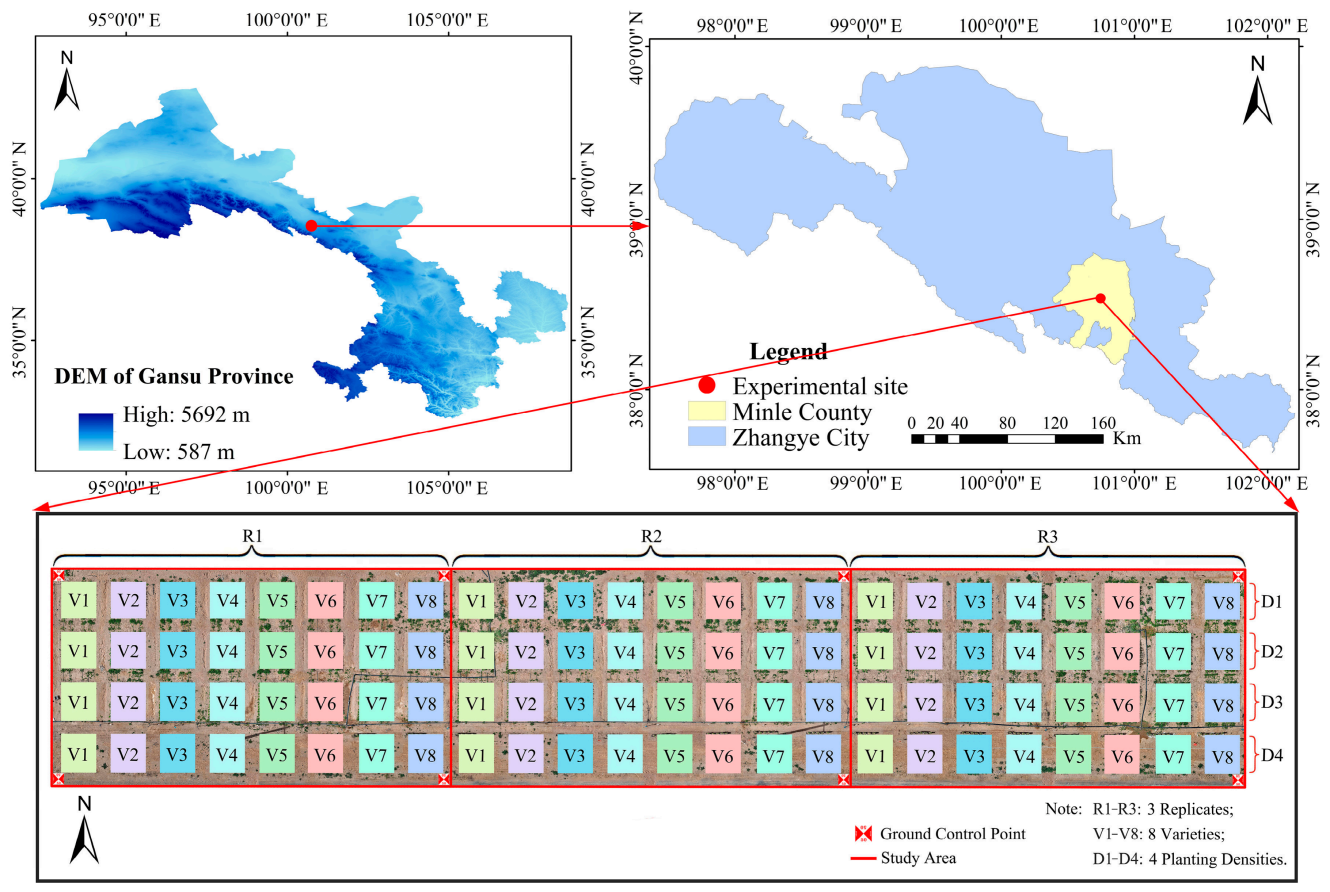
Overview of the study area.

The study area is located in Minle County, Zhangye City, Gansu Province (100°30′–101°00′ E, 38°20′–38°40′ N), situated in the middle reaches of the Hexi Corridor. Gansu is an important maize-producing region in northwestern China. In 2025, maize was cultivated on approximately 1.107 million ha, accounting for about 40% of the provincial grain-sown area and contributing approximately 55% of total grain production. Maize production systems vary across the province: irrigated agriculture is widely practiced in the Hexi Corridor and Yellow River irrigation zones, whereas rainfed dryland maize is common in central and eastern Gansu. The present study site in Minle County is located in the Hexi Corridor and represents a typical oasis agricultural environment. This region serves as a representative oasis agricultural zone (Figure 11). The topography generally slopes from south to north, characterized by the alluvial fan zone of the northern Qilian Mountains in the south and a diluvial plain in the north. The primary soil types are anthropogenic alluvial soil (irrigated desert soil) and Sierozem, which are conducive to the cultivation of dryland crops such as maize and wheat. The field experiment included eight maize hybrids (V1–V8), four planting-density treatments (D1–D4), and three replicate blocks (R1–R3). The specific maize hybrids are listed in Table 3. The planting densities were 60,000, 75,000, 90,000, and 105,000 plants ha^−1^ for D1, D2, D3, and D4, respectively. A uniform row spacing of 0.50 m was used across all treatments, and each experimental plot consisted of six maize rows. Within each replicate block, four planting-density treatment areas were established, and the eight maize hybrids were randomly arranged within each density treatment. Each hybrid–density combination was included once in each replicate. Therefore, each replicate contained 32 experimental plots, resulting in a total of 96 plots per year. The same experimental design, treatment arrangement, and replication scheme were adopted in both 2024 and 2025, resulting in 192 experimental plots across the two growing seasons. All maize hybrids were sown according to the same schedule within each growing season, and days after sowing (DAS) were used as the standardized temporal scale in all subsequent growth-curve and prediction analyses. Except for the planting-density treatments, irrigation, fertilization, and weed and pest management were applied consistently across all experimental plots. The experiment therefore covered two complete growing seasons. The 2024 dataset was used for prior growth-rate parameter estimation, whereas the 2025 dataset was used for cross-year prediction evaluation. The eight maize hybrids and four planting-density treatments were included to provide diverse plant-height growth trajectories for evaluating the applicability of the proposed framework under different growth conditions.

### 4.2. Data Sources and Preprocessing

To systematically evaluate the applicability and accuracy of different remote-sensing sensors for maize plant-height estimation, paired RGB imagery and LiDAR point-cloud data were acquired using UAV platforms during the 2024 and 2025 maize growing seasons. Twenty-five UAV observation campaigns were conducted in 2024, and 25 campaigns were conducted in 2025, covering the period from 7 to 137 days after sowing (DAS) in each growing season. The observations extended from 24 April to 1 September 2024 and from 27 April to 10 September 2025, covering maize development from the seedling to grain-filling stages. RGB imagery was acquired using a DJI Phantom 4 RTK (Shenzhen, China), whereas LiDAR point clouds were collected using a DJI Matrice 300 RTK (Shenzhen, China) equipped with a Zenmuse L1 sensor (Shenzhen, China).

#### 4.2.1. Acquisition and Preprocessing of Rgb Images

In this study, a DJI Phantom 4 RTK UAV, integrated with an onboard RTK module, was employed to collect low-altitude remote sensing imagery throughout the maize growth cycle from 2024 to 2025. Flight operations were conducted under clear and windless conditions with uniform illumination. The flight parameters were set to an altitude of 30 m, with 80% longitudinal and 70% lateral overlaps. The resulting RGB imagery achieved a ground sampling distance (GSD) of better than 3 cm/pixel, enabling clear identification of maize tassels, canopy textures, and row-plant spacing structures.

Three-dimensional (3D) modeling of the RGB imagery was performed using the Structure from Motion (SfM) algorithm. Following a quality assessment, the raw images were imported into Pix4Dmapper, which automatically retrieved internal camera parameters, distortion coefficients, and RTK positioning data for coordinate system initialization and initial registration. Feature points were extracted and matched using the SfM algorithm, followed by aerotriangulation to solve for exterior orientation elements. A global optimization of camera poses was conducted using bundle adjustment to minimize reconstruction errors and enhance spatial consistency. Based on the optimized poses, a high-density 3D point cloud was generated using Multi-View Stereo (MVS) algorithms, and a DSM was subsequently produced via Inverse Distance Weighting (IDW) interpolation.

#### 4.2.2. Lidar Data Acquisition and Preprocessing

This study utilized a DJI Matrice 300 RTK UAV equipped with a Zenmuse L1 LiDAR module to acquire low-altitude remote sensing data over a corn experimental field. This sensor integrates a LiDAR scanner, a high-precision Inertial Measurement Unit (IMU), and an RTK module, enabling the generation of DSM and DTM through an integrated workflow. Flight missions were planned using DJI Pilot 2 with the following parameters: a flight altitude of 30 m, a velocity of 1.4 m/s, a side overlap of 70%, and a repetitive scanning mode. During the flight, RTK was enabled, and raw IMU data were recorded, yielding source files including .LDR, .IMU, .RTK, .MRK, and .CLI formats.

The LiDAR data were processed using DJI Terra (version 5.3.0) software. The POS information, alongside IMU/GNSS data, was automatically retrieved to reconstruct high-precision 3D point clouds. Point cloud accuracy optimization and smoothing filters were applied to reduce cloud thickness and eliminate discrete noise. Ground point classification was executed by adjusting iterative parameters based on the local topography to automatically separate ground from non-ground points. A DTM was generated via interpolation based on the classified ground points, while the DSM was derived directly from the original point clouds. Finally, the point clouds were exported in LAS format, containing 3D coordinates, RGB values, and reflectivity information. Eight ground control points (GCPs) were established around and within the experimental field to ensure adequate spatial coverage. Their three-dimensional coordinates were measured using an RTK-GNSS receiver in fixed-solution mode. The GCPs were used for RGB image georeferencing and for verifying the spatial alignment of the LiDAR point cloud. The planimetric and vertical RMSEs were 0.028 m and 0.043 m, respectively.

#### 4.2.3. Field Data Collection

**Figure 12:**
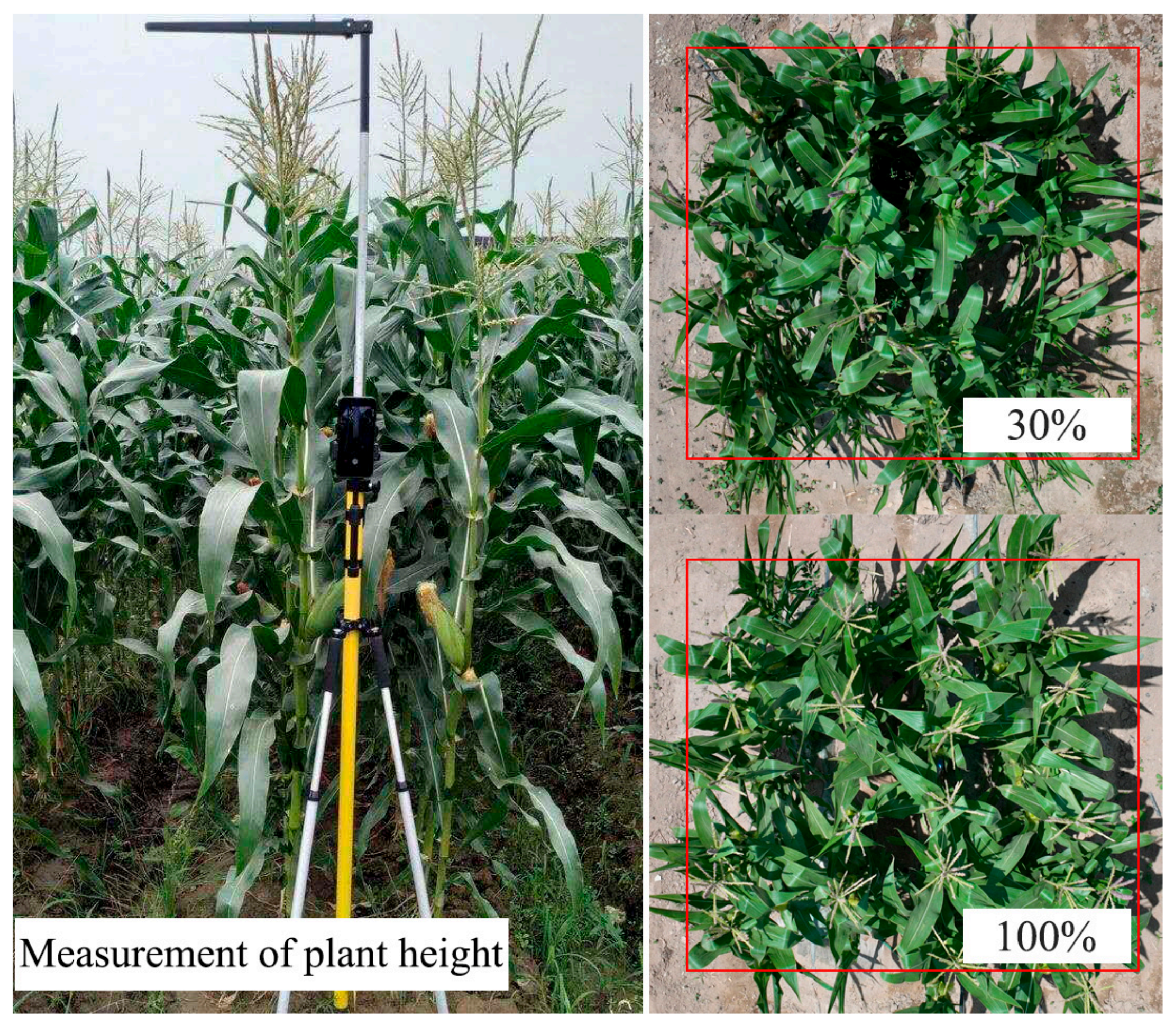
Field data acquisition.

Plant height was measured using a telescopic leveling rod as the vertical distance from the ground at the plant base to the highest natural point of the plant. Within each plot, three to six plants were measured in a predefined non-destructive sampling area located within the UAV remote-sensing region of interest (ROI). The measured plants had upright and intact stems, showed no visible lodging or mechanical damage, and were located away from the plot boundaries. The same selection criteria were applied across all plots and measurement dates. The mean height of the measured plants was used as the plot-level field reference, and the corresponding standard deviation was calculated to characterize within-plot variation (Figure 12, left). The tasseling stage was determined following the 50% phenological stage criterion widely adopted in agrometeorology and crop cultivation [26]. Based on high-resolution UAV RGB imagery, the tasseling proportion of each plot was calculated as the number of plants with visible tassels divided by the total number of plants within the plot. When the tasseling proportions on two consecutive observation dates were below and above 50%, respectively, the date of 50% tasseling (T_50_) was determined by linear interpolation. The mean interval between consecutive UAV observations was 9.7 d, ranging from 4 to 19 d. During the tasseling period, the observation interval ranged from 4 to 6 d, with a mean of approximately 4.7 d. Accordingly, the temporal uncertainty of the reference tasseling dates was estimated to be approximately ±2–3 d. The mean height of the sampled plants was defined as the field-measured plot-level mean plant height. Although the UAV-derived canopy height and the field-measured mean plant height differ in spatial scale and statistical meaning, canopy height was used as a remote-sensing proxy for plot-level mean plant height because both reflect the vertical growth of maize within the plot.

## 5. Materials and Methods

### 5.1. Principle of ROI Delineation

**Figure 13:**
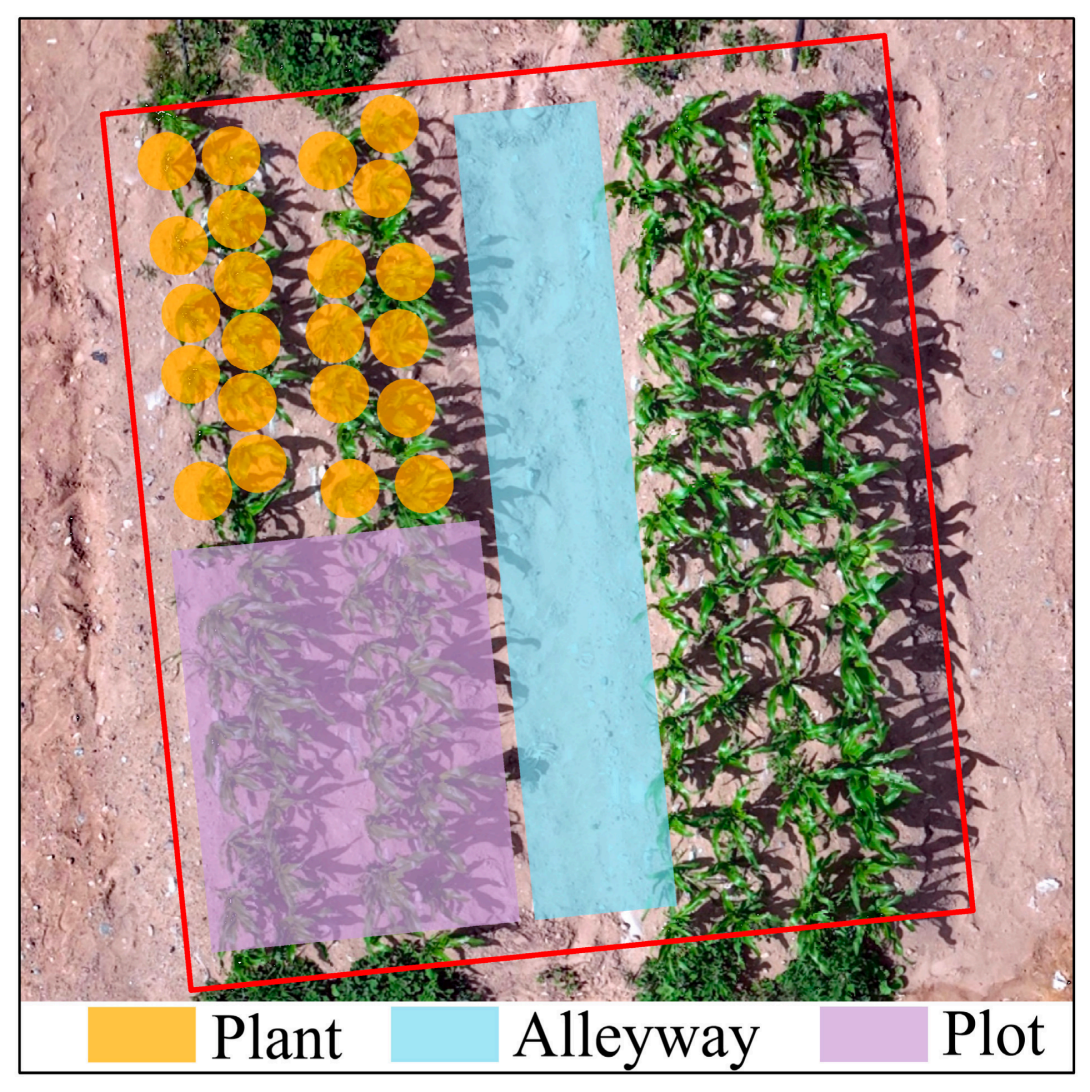
ROI selection strategy.

The delineation of the ROI critically determines the representativeness and accuracy of the statistical features extracted from the DSM and DTM. To systematically assess the effects of various ROI selection strategies on plant height estimation, this study devised a progressive ROI delineation method that expands outward from the interior and from vegetated to bare soil surfaces, as depicted in Figure 13. First, ROI boundaries were visually interpreted using digital orthophoto maps, adhering to the following principles: excluding only the designated destructive-sampling area on the right-hand side of each plot, while retaining the adjacent non-destructive plant-height sampling area within the remote-sensing ROI, shrinking each boundary inward by at least 0.5 m to mitigate edge effects, and excluding zones with missing seedlings, lodging, or weed infestation. This ROI design maintained the spatial correspondence between the field-sampling area and the UAV observations, while restricting both measurements primarily to the intact and non-lodged canopy portion of each plot. Second, 20–25 circular sub-windows were established within each ROI. The statistical features of the DSM were extracted from each sub-window, and their arithmetic mean was calculated as the representative canopy top elevation of the plot. Finally, the aisle was used to delineate a ground reference area, where DSM values directly represent ground elevation. Three spatial regions were used for feature extraction: the plot-level ROI, the plant subregions represented by 20–25 circular sub-windows within each plot, and the adjacent alleyway ground-reference region. Candidate canopy-top elevations were represented by the maximum and the 90th, 95th, and 99th percentiles of DSM values extracted from the plot-level ROI and plant subregions. Candidate ground elevations were represented by the minimum and the 5th and 10th percentiles of DTM values extracted from the plot-level ROI and the alleyway region. For each candidate combination, UAV-derived plant height was calculated as the selected DSM canopy elevation minus the selected DTM ground elevation. The resulting estimates were compared with field-measured plot-level plant height, and RMSE was calculated for each combination. These values served to verify the stability and reliability of the LiDAR-derived DTM under fully canopy-covered conditions. ROI delineation therefore followed a semi-manual workflow. Plot boundaries were visually interpreted from the orthomosaic according to predefined and consistent criteria, while the subsequent extraction and calculation of DSM and DTM features followed a standardized procedure. The predefined criteria helped reduce operator-related variability. The field reference represented the mean height of three to six plants measured within the non-destructive sampling area, whereas the UAV-derived metric summarized the upper-canopy structure over the plot-level ROI. Because the field-sampling area was retained within the UAV ROI and areas affected by lodging, missing plants, weeds, and destructive sampling were excluded, the two observations primarily characterized the same intact canopy domain. The selected UAV metric was therefore interpreted as an empirical plot-level upper-canopy proxy for tracking temporal plant-height dynamics. High-percentile canopy elevation metrics were selected because they retained the dominant upper-canopy information while reducing the influence of isolated extreme values.

### 5.2. Plant Height Time-Series Reconstruction and Logistic Model

The Logistic model is a nonlinear growth function extensively utilized in biology, ecology, and agronomy. It has garnered significant attention due to its capacity to effectively describe the sigmoidal growth patterns of organisms within resource-limited environments [27]. Originally proposed by the Belgian mathematician Pierre François Verhulst in 1838 and subsequently popularized by Pearl and Reed in demographic studies, this model has evolved into a vital tool for simulating population dynamics and individual development [28]. In crop science, the Logistic model is widely employed to fit the temporal variations of agronomic parameters, such as plant height, leaf area index, and dry matter accumulation, as its curve morphology aligns closely with the growth rhythms throughout the crop lifecycle [29]. Before growth-curve fitting, the UAV-derived height observations of each plot were arranged chronologically according to days after sowing. Under normal and non-lodged conditions, maize height was assumed to exhibit a generally non-decreasing trend. This assumption was applied only to the cumulative height trajectory of plots without visible lodging or structural canopy damage and did not imply that the growth rate remained continuously positive. A gradual decrease in growth rate or the cessation of height growth was therefore retained as a biologically valid pattern. Regions with visible lodging were excluded during ROI delineation. Temporary decreases in UAV-derived canopy height may arise from wind-induced canopy movement, changes in leaf orientation, or measurement uncertainty and were therefore treated as potential anomalies rather than being removed automatically. An observation was provisionally flagged when its height was more than 0.15 m lower than the preceding valid observation. This threshold was used as a conservative operational quality-control criterion to distinguish abrupt decreases from minor short-term fluctuations and was set above the vertical georeferencing uncertainty reported in Section 2.2. For consecutive decreases, each observation was compared with the most recent valid value. Only temporary decreases followed by subsequent height recovery were treated as potential measurement anomalies, whereas persistent decreases without recovery were retained because they could reflect actual canopy changes caused by lodging or environmental disturbance.

A preliminary Logistic model was then fitted to the retained observations using nonlinear least squares. The standardized residual was calculated as $z_{i}=(y_{i}-{\hat{y}}_{\iota })/s_{e}$, where $y_{i}$ and ${\hat{y}}_{\iota }$ are the observed and fitted plant heights, respectively, and $s_{e}$ is the standard deviation of the residuals from the current fit. Model fitting and residual screening were repeated until no new observations were flagged or a maximum of five iterations was reached. The five-iteration setting was used only as an upper stopping safeguard to prevent repeated fitting rather than as an outlier-identification criterion. Missing and flagged observations were reconstructed using the final fitted curve at the corresponding observation dates, whereas valid original observations were retained.

The fundamental mathematical form of the Logistic model is typically expressed as a function of growth increment over time. Following time-series reconstruction, a Logistic function was used to fit the UAV-derived height growth curve of each experimental plot. Model parameters were estimated using the nonlinear least squares method. Through iterative optimization, the sum of squared residuals between the fitted curves and measured values was minimized, thereby obtaining the optimal fitting curves for plant height growth. This provides a mathematical foundation for the subsequent extraction of tasseling stage feature points and the analysis of growth dynamics. The specific expression is as follows:

(1) $$\mathrm{MH}(t)=\frac{MH_{\mathrm{Max}}\;\times MH_{0}\times e^{r(t-t_{0})}}{MH_{\mathrm{Max}}+MH_{0}\times (e^{r(t-t_{0})}-1)}$$

where *t* denotes the observation time, expressed as days after sowing (DAS); *MH*(*t*) represents the maize plant height at time *t*; *MH*_0_ and *t*_0_ are the initial observed plant height and its corresponding DAS, respectively; *r* is the growth rate parameter, reflecting the steepness of the growth curve; and *MH_Max_* is the upper limit of plant height, representing the maximum height attained in the plot.

### 5.3. Gompertz Model

The Gompertz model is a classic non-linear growth fitting model widely utilized in research areas such as crop growth, biomass accumulation, plant height dynamics, and population changes [30], primarily due to its robust capacity to describe sigmoidal growth processes. Originally proposed by British mathematician Benjamin Gompertz in 1825, the model is founded on the core hypothesis that the growth rate decays exponentially over time. This allows it to accurately characterize the typical growth trajectory-starting with an initial slow growth phase, followed by a rapid mid-term acceleration, and concluding with a late-stage stabilization. Such a pattern aligns closely with the actual physiological rhythms of crops like maize, including plant height and dry matter accumulation [21]. Distinctively, the Gompertz model features an asymmetric S-shaped curve with an inflection point occurring at approximately 36.8% of its asymptotic limit. This characteristic is consistent with the empirical observation that crops exhibit a gradual start, rapid elongation, and eventual deceleration toward maturity, offering significant advantages in both fitting precision and biological interpretability [31]. The fundamental expression of the Gompertz model is as follows:

*MH*(*t*) = *A*·*exp*(−*B*·*exp*(−*C*·*t*)) (2)

where *MH*(*t*) is the observed maize plant height at days after sowing t; *A* is the growth limit, representing the theoretical maximum plant height attainable for the plot; *B* is a location parameter related to the starting point of growth; and *C* is the growth rate parameter, governing the timing of the curve’s inflection point and the duration of the rapid growth phase.

### 5.4. Feature Point Extraction

Maize tasseling marks the end of the vegetative growth phase, after which the growth rate of plant height gradually declines and eventually ceases. Based on this biological pattern, the fitted maize plant-height curves were differentiated to characterize temporal changes in growth rate, acceleration, and deceleration. After curve fitting and spline smoothing, the continuous plant-height curve was resampled at equal temporal intervals. Let MH_i_ = MH(t_i_) denote the fitted plant height at time t_i_, and let Δt denote the temporal interval. The first-, second-, and third-order derivatives, curve curvature, and normalized growth rate were calculated using central finite differences as follows:

(3) $$MH_{i}^{\prime }=\frac{MH_{i+1}-{MH}_{i-1}}{2\Delta t}$$

(4) $$MH_{i}^{\prime \prime }=\frac{MH_{i+1}-{{2MH}_{i}+MH}_{i-1}}{\Delta t^{2}}$$

(5) $$MH_{i}^{\prime \prime \prime }=\frac{MH_{i+2}-{{2MH}_{i+1}+{2MH}_{i-1}-MH}_{i-2}}{2\Delta t^{3}}$$

(6) $$k_{i}=\frac{MH^{\prime \prime }}{{\left[1+{\left(MH_{i}^{\prime }\right)}^{2}\right]}^{3/2}}$$

(7) $$v_{norm}(t)=\frac{MH^{\prime }(t)-MH_{min}^{\prime }}{MH_{max}^{\prime }-MH_{min}^{\prime }}$$

where $MH_{i}^{\prime }$, $MH_{i}^{\prime \prime }$ and $MH_{i}^{\prime \prime \prime }$ represent the first-, second-, and third-order derivatives at time *t_i_*, respectively; *k_i_* is the signed curvature; $MH_{max}^{\prime }$ and $MH_{min}^{\prime }$ are the maximum and minimum values of the first derivative; and $v_{norm}(t)$ is the normalized growth rate.

**Figure 14:**
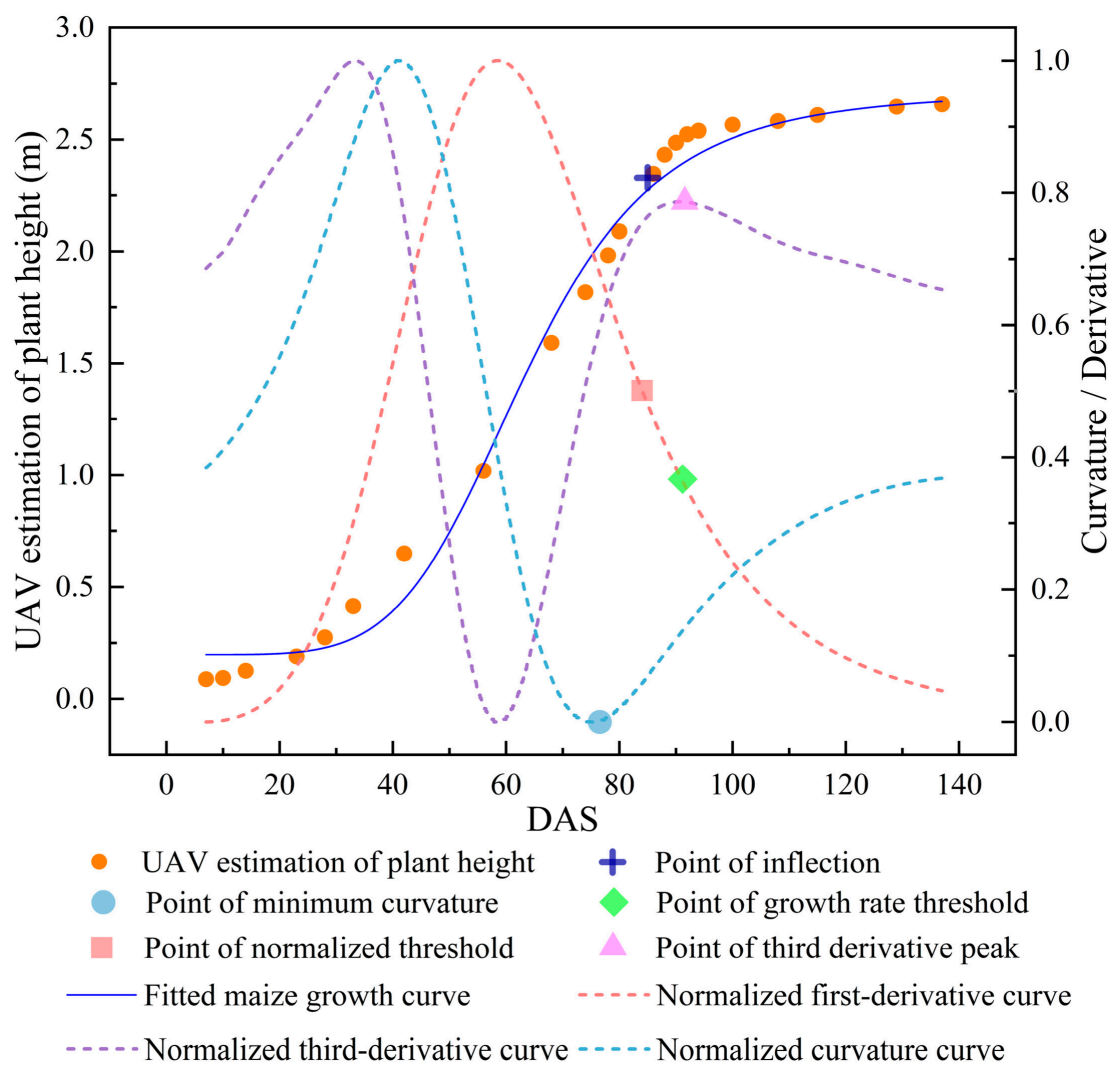
Identification of maize tasseling stage based on plant height time series.

Five characteristic points associated with the transition from rapid growth to growth stagnation were extracted as follows (Figure 14): (1) POI: After spline smoothing, the point of maximum curvature change in the first-difference sequence of plant height was identified, corresponding to the critical moment when the plant height shifts from rapid to slow growth. (2) Point of minimum curvature: The local minimum on the curvature curve, indicating the most flattened portion of the growth curve where plant growth has essentially ceased, reflecting the entry into vegetative stagnation. (3) Point of growth rate threshold: The instantaneous height growth rate was calculated from the first derivative of the fitted plant-height curve. On the descending branch after the maximum growth rate, the first time point at which the growth rate decreased to or below the predefined threshold of 0.02 m day^−1^ was identified as the growth-rate threshold point, indicating that maize height growth was approaching cessation. (4) To reduce the influence of differences in growth-rate magnitude among plots, the first derivative was transformed using min–max normalization. On the descending branch after the maximum growth rate, the first time point at which the normalized growth rate decreased to or below 0.5 was identified as the normalized threshold point. The same threshold criteria were applied to all experimental plots. (5) Point of third derivative peak: The time point at which the third derivative reaches a local maximum, where the deceleration process exhibits its maximal rate of change, signaling the transition of the growth rate decline from rapid to gradual, with growth gradually approaching stagnation. Together, these five characteristic points quantify the critical state marking the end of vegetative growth from different perspectives, providing a multi-indicator synthetic basis for determining the tasseling stage.

During tasseling-stage prediction, the available early-stage plant-height observations were fitted using the Logistic model, with the growth-rate parameter fixed to the prior value derived from the source-year data and the maximum plant height estimated separately for each plot. The fitted curve was then extrapolated over the subsequent growth period, and the predicted tasseling time was defined as the point of inflection extracted from the extrapolated curve using the same feature-point identification procedure described above. The predicted time was first expressed as days after sowing and then converted to the corresponding calendar date based on the sowing date.

### 5.5. Assessment of Model Predictive Accuracy

To evaluate the predictive performance of the models, three complementary metrics, namely the coefficient of determination (R^2^), root mean square error (RMSE), and mean absolute error (MAE), were used. R^2^ measures the proportion of variation in the observed values explained by the predicted values and therefore reflects goodness of fit. RMSE reflects the overall magnitude of prediction errors and assigns greater weight to relatively large deviations, whereas MAE represents the average absolute deviation between predicted and observed values and is less sensitive to individual large errors. Considering the objectives of this study, the combined use of R^2^, RMSE, and MAE provides an adequate basis for evaluating model accuracy and comparing different variable combinations, sensing modalities, growth-curve models, and tasseling-stage prediction results. The calculation formulas are as follows:

(8) $$R^{2}=1-\frac{\sum_{i=1}^{n}{{(y}_{i}-{\hat{y}}_{\iota })}^{2}}{\sum_{i=1}^{n}{{(y}_{i}-\bar{y})}^{2}}$$

(9) $$RMSE=\sqrt{\frac{1}{n}\sum_{i=1}^{n}{{(y}_{i}-{\hat{y}}_{\iota })}^{2}}$$

(10) $$MAE=\frac{1}{n}\sum_{i=1}^{n}\left|{(y}_{i}-{\hat{y}}_{\iota }\right|$$

where $y_{i}$ is the observed value of the i-th sample, ${\hat{y}}_{\iota }$ is the predicted value of the i-th sample, $\bar{y}$ is the mean of the observed values, and n is the total number of samples.

## 6. Conclusions

This study used UAV LiDAR remote sensing data to obtain time series of maize plant height across the full growth period from 2024 to 2025. The Logistic growth curve model was applied to fit the dynamic changes in plant height, providing a comprehensive investigation of the feasibility and accuracy of identifying and forecasting the maize tasseling stage based on the plant height growth curve. The results showed the following:

(1) Sensor selection significantly influenced the choice of variables for plant height estimation. For the LiDAR sensor, using the 99th percentile of the digital surface model extracted within the plot-based ROI to represent the canopy top yielded the best performance. For the RGB sensor, using the maximum DSM value extracted within the plot-based ROI to represent the canopy top elevation performed best, combined with the minimum DTM value extracted within the plot-based ROI, achieving an RMSE as low as 0.29 m.
(2) Based on high-accuracy plant height time series data, the fitting performances of the Logistic and Gompertz growth curves were compared, and the Logistic model was determined to be more effective in describing the dynamic changes in maize plant height. The results showed that the inflection point achieved the highest accuracy in tasseling stage identification, with R^2^ of 0.77, RMSE of 2.478 d, and MAE of 2.124 d for the raw time series. For the recovered time series, R^2^ remained at 0.75, with RMSE of 2.586 d and MAE of 2.338 d.
(3) Using early plant height time series data, the Logistic growth function along with preset growth rate parameters was employed to dynamically simulate the growth trend of maize plant height. The inflection point feature was then extracted from the fitted curve to enable effective forecasting of the tasseling stage. The results showed that prediction accuracy improved significantly as the plant height threshold increased, with the prediction RMSE for plant height gradually decreasing from 0.543 m to 0.342 m. When the plant height threshold was ≥1.6 m and the growth rate ranged between 0.11 and 0.13, the tasseling stage forecast achieved optimal performance, with an RMSE stabilized between 3.0 and 4.0 d for the raw time series.

## 7. Research Limitations and Prospects

This study systematically investigated the construction of maize plant height time series across the full growth period, growth curve fitting, and tasseling stage identification and early prediction based on UAV LiDAR remote sensing data. The feasibility and efficacy of predicting key phenological stages from plant height dynamics were validated, providing methodological support for crop phenotyping and precision management. However, the following limitations warrant further investigation.

(1) Our method relies on plant height data from the full growth period, with prediction initiation requiring plant height to reach a specific threshold, which limits lead time. Moreover, insufficient early season data exacerbates the uncertainty of model extrapolation. Future efforts could incorporate historical data from the same region or growth parameters of similar varieties from neighboring areas as prior knowledge, combined with transfer learning, to further advance the prediction initiation window.
(2) Reconstruction processing had a limited effect on accuracy in full-growth-period identification and even resulted in a slight decrease in early prediction accuracy. Moreover, the monotonic reconstruction method was developed for non-lodged maize canopies, and its robustness under persistent height loss caused by severe lodging or environmental stress was not independently evaluated. Future studies should explore adaptive reconstruction strategies that preserve the original growth characteristics while excluding obvious outliers and should further validate the method under abnormal growth conditions.
(3) The plot-level height metric was calculated from high-percentile DSM and minimum DTM values, which may originate from different locations and introduce bias under uneven terrain. Future studies will compare this method with pixel-wise CHM, local ground normalization, and standard CHM percentile approaches.
(4) Although cross-year evaluation was conducted using data from the 2024 and 2025 growing seasons, both experiments were carried out at the same site, and the model comparison was limited to Logistic, Gompertz, and the proposed framework without quantitative benchmarking against deep-learning, Transformer-based, other temporal sequence models, or additional nonlinear growth functions. Future studies will use larger multi-region, multi-year, and multi-environment datasets to further evaluate model generalization and systematically compare Richards, Weibull, Von Bertalanffy, machine-learning regression, and deep temporal models in terms of prediction accuracy, data requirements, and interpretability.
(5) Model performance in this study was evaluated using R^2^, RMSE, and MAE, which provided complementary measures of goodness of fit, overall error magnitude, and average absolute deviation and adequately addressed the accuracy-assessment objectives of the present study. Future studies will further extend the evaluation framework by incorporating confidence intervals, paired statistical tests, Bland–Altman analysis, error-distribution analysis, and normalized performance metrics to more comprehensively characterize prediction uncertainty and agreement among sensing modalities.

## Figures and Tables

**Table 1:** Distribution of fitted parameters from the Logistic model.

	Plant Height Growth Rate (d^−1^)			Maximum Plant Height (m)		
	Range	Average	Standard Deviation	Range	Average	Standard Deviation
Raw	0.0717–0.1677	0.1189	0.0244	2.086–2.924	2.537	0.226
Recovered	0.0701–0.1523	0.1180	0.0184	2.137–3.012	2.670	0.224

**Table 2:** Error assessment of maize tasseling stage identification results.

	Point of Inflection		Point of Minimum Curvature		Point of Growth Rate Threshold		Point of Normalized Threshold		Point of Third Derivative Peak	
	RMSE	MAE	RMSE	MAE	RMSE	MAE	RMSE	MAE	RMSE	MAE
Raw	2.478	2.124	3.909	3.283	5.961	5.384	5.897	5.101	4.385	3.643
Recovered	2.586	2.338	5.356	4.637	4.812	3.969	6.157	5.498	3.542	2.890

**Table 3:** Maize cultivars used in the experiments.

Number	Variety Name	Number	Variety Name
V1	Zhengdan 958	V5	Nongda 108
V2	Xianyu 335	V6	Yinong 103
V3	Denghai 661	V7	Zhongdan 909
V4	Jingnongke 728	V8	Nonghua 101

## Data Availability

The datasets generated and/or analyzed during the current study are not publicly available due to the limited availability of some data but are available from the corresponding author on reasonable request.
